# Association of pre-transplant statin use with delayed graft function in kidney transplant recipients

**DOI:** 10.1186/1471-2369-13-111

**Published:** 2012-09-17

**Authors:** Janske Reiling, David W Johnson, Peter S Kruger, Peter Pillans, Daryl R Wall

**Affiliations:** 1 Queensland Renal Transplant Service, Princess Alexandra Hospital, Brisbane, Australia; 2 Maastricht University, Maastricht, The Netherlands; 3 University of Queensland, Brisbane, Australia; 4 Intensive Care Unit, Princess Alexandra Hospital, Brisbane, Australia; 5 Pharmacology Department, Princess Alexandra Hospital, Brisbane, Australia

## Abstract

**Background:**

Administration of HMG-CoA reductase inhibitors (statins), prior to ischemia or prior to reperfusion has been shown to decrease ischemia-reperfusion renal injury in animal studies. It is unknown whether this protective effect is applicable to renal transplantation in humans. The aim of this study was to determine the relationship between prior statin use in renal transplant recipients and the subsequent risk of delayed graft function.

**Methods:**

All patients who underwent deceased or living donor renal transplantation at the Princess Alexandra Hospital between 1 July 2008 and 1 August 2010 were included in this retrospective, observational cohort study. Graft function was classified as immediate graft function (IGF), dialysis-requiring (D-DGF) and non-dialysis-requiring (ND-DGF) delayed graft function. The independent predictors of graft function were evaluated by multivariable logistic regression, adjusting for donor characteristics, recipient characteristics, HLA mismatch and ischaemic times.

**Results:**

Overall, of the 266 renal transplant recipients, 21% exhibited D-DGF, 39% had ND-DGF and 40% had IGF. Statin use prior to renal transplantation was not significantly associated with the risk of D-DGF (adjusted odds ratio [OR] 1.05, 95% CI 0.96 – 1.15, P = 0.28). This finding was not altered when D-DGF and ND-DGF were pooled together (OR 0.98; 95% CI 0.89-1.06, p = 0.56).

**Conclusions:**

The present study did not show a significant, independent association between prior statin use in kidney transplant recipients and the occurrence of delayed graft function.

## Background

Delayed graft function (DGF), describing impairment of graft function immediately after transplantation, is associated with significant morbidity, including increased risks of acute allograft rejection, prolonged hospitalization, higher health care costs and poorer graft survival [1-6]. The incidence of DGF in the renal transplant population varies from as low as 4.7% in live related transplants [7] to as high as 53-69% in kidneys following donation after cardiac death (DCD) [8]. Factors associated with an increased risk of DGF include both recipient factors (male gender, pre-transplant diabetes mellitus, increased BMI, greater HLA mismatch, higher panel reactive antibodies, previous blood transfusions, previous transplants, pre-transplant dialysis) and donor factors (older age, anoxia, cerebrovascular accident, hypertension, deceased donor, donation after cardiac death [DCD], longer cold ischemic time and higher terminal serum creatinine concentration) [9-12].

DGF is strongly associated with longer periods of ischemia between retrieval of the kidney from the donor and subsequent reperfusion of the kidney in the recipient. Such ischemic injury tends to be more marked in deceased donor renal transplantation, particularly donation after cardiac death [8]. The reintroduction of renal blood flow is associated with the production of oxygen free radicals, which in turn promote inflammation, necrosis, and apoptosis within the renal allograft [13,14]. Although there are currently no treatments that effectively reduce the severity of ischemia-reperfusion injury and delayed graft function, 3-hydroxymethylglutaryl coenzyme A inhibitors (also known as statins) show considerable promise. In addition to lowering serum cholesterol, these agents decrease the formation of reactive oxygen species and inflammatory cytokines by inhibiting the isoprenylation of intracellular signal molecules (Ras, Rac1, cdc42 and Rho), so-called pleiotropic effects [15]. Administration of statins prior to ischemia or prior to reperfusion has been shown to decrease ischemia-reperfusion renal injury in rats [16-22]. However, it is unknown whether this protective effect is applicable to renal transplantation in human beings.

The aim of this study was to determine the relationship between prior statin use in renal transplant recipients and the subsequent risk of DGF.

## Methods

All patients who underwent deceased or living donor renal transplantation at the Princess Alexandra Hospital between 1 July 2008 and 1 August 2010 were included in this retrospective, observational cohort study. All T cell cross matches were negative. The preservation fluid used was University of Wisconsin preservation solution. An interleukin-2 receptor antagonist (basiliximab) was routinely administered at induction of immunosuppression. The immunosuppression regimen included a calcineurin inhibitor (primarily tacrolimus), prednisolone and mycophenolate mofetil. Tacrolimus dosages were titrated to maintain trough serum concentrations between 8 and 10 μg/L. Cyclosporine was used in a small minority of patients with a low immunological risk or if there was a contra-indication for tacrolimus use. All anti-hypertensive agents were ceased prior to transplantation and avoided during the first two post-operative weeks. Dopamine and other inotropic agents were not administered to any recipient during the study period.

### Data collection

Data collection for the study was approved by the Princess Alexandra Hospital Research Ethics Committee and individual consent was obtained from all transplant recipients. For each patient, demographic data, operative data, donor data, post-operative complications, medical complications, admission histories, medications and renal allograft function were prospectively recorded on a computerised integrated renal database. If recipients were using statins prior to transplantation, the type and dose of statin were recorded. Unfortunately there were no data available concerning the duration of statin use prior to transplantation.

### Classification and outcome measure

Graft function after transplantation was classified as dialysis- delayed graft function (D-DGF) when recipients required dialysis within the first 72 h post transplantation, non-dialysis delayed graft function (ND-DGF) [23,24] when the creatinine reduction ratio at post-operative day 2 (CRR2) was less than 30% without the need for dialysis and immediate graft function (IGF) when the CRR2 value was greater than 30%. The CRR2 was calculated using the creatinine levels on post operative days 1 (Cr1) and 2 (Cr2) using the following formula:

$$\text{CRR}2\left(\%\right)=\left(\left[\text{Cr}1–\text{Cr}2\right]\times 100\right)/\text{Cr}1$$

The primary outcome measure was the incidence of delayed graft function ( both D-DGF and ND-DGF were assessed).

### Statistical analysis

Results are presented as number (%) for categorical data, mean ± SD for continuous data and median (interquartile range; 25^th^-75^th^ percentile) for continuous variables not normally distributed. Comparisons between groups were made by χ^2^ test for categorical variables, unpaired t-test for continuous normally distributed variables and Mann–Whitney test for continuous variables not normally distributed. The independent predictors of DGF were evaluated by multivariable logistic regression using backward stepwise elimination based on a p value cut-point of 0.2 until the most parsimonious model was identified. Variables initially included in the model were donor characteristics (age, gender, body mass index [BMI], hypertension, diabetes mellitus, smoking status, donor type, cause of death, inotropic support, estimated glomerular filtration rate [eGFR]), recipient characteristics (age, gender, race, BMI, hypertension, cardiovascular disease, diabetes mellitus, smoking status, end-stage renal failure cause, prior renal replacement therapy, previous renal transplantation) and operation characteristics (number of HLA mismatches, cold ischemic time, warm ischemic time). BMI was categorized as underweight (<18.5 kg/m^2^), normal range (18.5-24.99 kg/m^2^), overweight (25–29.99 kg/m^2^) and obese (>30 kg/m^2^) according to the World Health Organization [25].

First-order interaction terms between the significant covariates were examined for all models. Data were analysed using the software packages SPSS for Windows, Release Version 18.0, (© SPSS, Inc., 2009, Chicago, IL, http://www.spss.com). P values less than 0.05 were considered statistically significant. No assumptions were made regarding missing data and all proportions were calculated as percentages of the patients with available data.

## Results

### Population characteristics

A total of 270 patients underwent renal transplantation during the study period. 269 patients were included in the final analysis as prior statin use could not be determined in one recipient. Of these, 93 (35%) patients were using statins prior to transplantation. Compared with patients not receiving statins, prior statin users were significantly more likely to be older, hypertensive and a lower degree of HLA mismatch with the donor. They also tended to have a higher BMI, a history or prior renal transplantation and a kidney from an older donor (Table 1). There were no statistically significant differences between the two groups with respect to presence of cardiovascular disease, diabetes mellitus or any of the other donor, recipient or operative characteristics listed in Table 1.

**Table 1:** Baseline recipient, donor and transplant procedure characteristics

**Characteristics**	**Statin**	**Non-statin**	**Total**	**p-value**
	**N = 93**	**N = 176**	**N = 269**	
**Recipient characteristics**				
Age (years)	53.7 ± 11.0	47.5 ± 13.5	49.7 ± 13.0	<0.001
Male gender (%)	62(67%)	115(65%)	177(66%)	0.83
Race				0.06
Caucasian	81(87%)	150(85%)	231(86%)	
Other	12(13%)	26(15%)	38(14%)	
Body Mass Index^1^ (kg/m^2^)				0.08
<18,5	1(1%)	5(3%)	6(2%)	
18.5-24.9	33(37%)	76(43%)	109(41%)	
25-29.9	32(35%)	70(40%)	102(38%)	
>30	24(27%)	25(14%)	49(19%)	
Co-morbidities				
Hypertension	89(96%)	148(84%)	237(88%)	0.005
Coronary artery disease	11(12%)	12(7%)	23(9%)	0.16
Peripheral vascular disease	4(4%)	5(3%)	9(3%)	0.53
Cerebrovascular disease	2(2%)	2(1%)	4(1%)	0.51
Chronic lung disease	6(7%)	12(7%)	18(7%)	0.91
Diabetes mellitus	9(10%)	12(7%)	21(8%)	0.41
Smoker				0.78
Current	1(1%)	1(1%)	2(1%)	
Former	41(44%)	84(48%)	125(46%)	
Never	51(55%)	91(51%)	142(53%)	
Etiology of ESRD				0.18
Polycystic kidney disease	23(25%)	30(17%)	53(19%)	
Glomerulonephritis	14(15%)	33(19%)	47(17%)	
Focal sclerosing	4(4%)	6(3%)	10(4%)	
Focal and segmental proliferative	0(0%)	9(5%)	9(3%)	
IgA nephropathy	14(15%)	29(16%)	43(16%)	
Reflux nephropathy	8(9%)	19(11%)	27(10%)	
Diabetic nephropathy	7 (8%)	4 (2%)	11 (4%)	
Other	27(29%)	61(35%)	88(33%)	
Renal replacement therapy				0.55
Haemodialysis	56(60%)	97(55%)	153(57%)	
Peritoneal dialysis	27(29%)	52(30%)	79(29%)	
None	10(11%)	27(15%)	37(14%)	
Previous transplants	15%	9%	11%	0.10
Length of admission (days)	8.6 ± 4.2	7.9 ± 3.8	8.2 ± 4.0	0.19
**Donor characteristics**				
Age at transplant (years)	47.8 ± 14.6	44.0 ± 15.2	45.33 ± 13.02	0.05
Male gender (%)	42(45%)	83(47%)	125(47%)	0.76
Body Mass Index^1^ (kg/m^2^)				0.23
<18.5	0(0%)	8(5%)	8(3%)	
18.5-24.9	32(35%)	57(33%)	89(34%)	
25-29.9	42(46%)	76(43%)	108(45%)	
>30	17(19%)	32(19%)	49(18%)	
Co-morbidities				
Hypertension	14(15%)	20(11%)	34(13%)	0.38
Hypertension	14(15%)	20(11%)	34(13%)	0.38
Diabetes	2(2%)	4(2%)	6(2%)	0.83
Current	23(25%)	49(28%)	72(27%)	
Former	21(22%)	36(20%)	56(21%)	
Never	49(53%)	91(52%)	140(52%)	
Donor type				0.35
Donation after brain death	49(53%)	94(53%)	143(53%)	
Donation after cardiac death	13(14%)	15(9%)	28(10%)	
Life	31(33%)	67(38%)	98(37%)	
Cause of death	0.36			
Subarachnoid haemorrhage	22(36%)	28(26%)	50(29%)	
Cardiac arrest	6(10%)	19(17%)	25(15%)	
Intracranial haemorrhage	9(14%)	15(14%)	24(14%)	
Cyclist	6(10%)	4(4%)	10(6%)	
Fall	4(6%)	5(4%)	9(5%)	
Traffic accident	2(3%)	5(4%)	7(4%)	
Hypoxia	3(5%)	4(4%)	7(4%)	
Other	10(16%)	29(27%)	39(23%)	
Inotropic support	52(84%)	95(87%)	147(86%)	0.55
eGFR (mL/min/1.73 m^2^)	90.8 ± 22.8	94.4 ± 25.0	93.2 ± 24.3	0.25
**Transplant procedure characteristics**				
Mismatch	3(2–5)	4(2–5)	4(2–5)	0.05
Cold ischaemic time (hours)	8.2 ± 4.5	7.6 ± 4.5	7.7 ± 4.5	0.29
Warm ischaemic time (hours)	0.6 ± 0.2	0.6 ± 0.2	0.6 ± 0.2	0.93

^1^BMI classification according to the WHO.

The majority (77%) of recipients using statins prior to transplantation were prescribed atorvastatin followed by simvastatin (14%), pravastatin (8%) and rosuvastatin (1%). 97% of the atorvastatin users used a daily dose of 40 mg or less (Table 2).

**Table 2:** Types of statins used in recipients

**Statin type**	**Frequency (N = 93)**	**Percentage of total**
**Atorvastatin**		
10 mg	21	22.6%
20 mg	29	31.2%
40 mg	20	21.5%
80 mg	2	2.2%
** Total**	**72**	**77.4%**
**Pravastatin**		
20 mg	3	3.2%
40 mg	4	4.3%
** Total**	**7**	**7.5%**
**Simvastatin**		
5 mg	1	1.1%
10 mg	3	3.2%
20 mg	8	8.6%
40 mg	1	1.1%
** Total**	**13**	**14.0%**
**Rosuvastatin**		
40 mg	1	1.1%
** Total**	**1**	**1.1%**

### Graft function after transplantation

Table 3 shows graft function after transplantation. The CRR2 of 3 patients could not be calculated and were therefore excluded from the analysis. Overall, 57 (21%) of renal transplant recipients exhibited D-DGF, 103 (39%) exhibited ND-DGF and 106 (40%) exhibited IGF. The occurrence of D-DGF was comparable between recipients who did and did not use statins prior to renal transplantation (21 (23%) versus 36 (21%), respectively, p = 0.69). The occurrence of ND-DGF was not significantly different between statin users and non-statin users (40 (43%) versus 63(36%), p = 0.25). When we pooled D-DGF and ND-DGF, the overall rate of DGF was not significantly different between statin users and non-statin users (61 (66%) versus 99(57%), respectively, p = 0.14). The rates of pooled D-DGF and ND-DGF were comparable between patients using atorvastatin and those using other statins (38 (66%) versus 23(68%), respectively, p = 0.32).

**Table 3:** Graft function after transplantation

**Donor type**	**Graft function**	**Statin**	**Non-statin**	**Total**	**p-value**
		**N = 92**	**N = 174**	**N = 266**	
All					0.32
	IGF	31(34%)	75(43%)	106(40%)	0.14
	ND-DGF	40(43%)	63(36%)	103(39%)	0.25
	D-DGF	21(23%)	36(21%)	57(21%)	0.69
Donation after Cardiac Death		N = 13	N = 15	N = 28	0.23
	IGF	0(0%)	0(0%)	0(0%)	
	ND-DGF	4(31%)	8(53%)	12(43%)	
	D-DGF	9(69%)	7(47%)	16(57%)	
Donation after Brain Death		N = 49	N = 94	N = 143	0.80
	IGF	15(31%)	27(29%)	42(29%)	
	ND-DGF	23(47%)	41(43%)	64(45%)	
	D-DGF	11(22%)	26(28%)	37(26%)	
Living Donor		N = 30	N = 65	N = 95	0.09
	IGF	16(54%)	48(74%)	64(68%)	
	ND-DGF	13(43%)	14(21%)	27(28%)	
	D-DGF	1(3%)	3(5%)	4(4%)	

IGF, Immediate Graft Function; ND-DGF, Non Dialysis dependent Delayed Graft function; D-DGF, Dialysis dependent Delayed Graft Function.

The rates of acute rejection were not significantly different between recipients using statins (n = 16, 17%) and those who did not (n = 17, 10%; p = 0.17).

### Delayed graft function occurrence according to statin usage

Using multivariable logistic regression analysis, D-DGF was not significantly associated with statin use (adjusted odds ratio [OR] 1.05, 95% CI 0.96 – 1.15 p = 0.28) (Table 4). Clustered D-DGF + ND-DGF was also not significantly associated with statin usage (OR 0.98; 95% CI 0.89-1.06, p = 0.56) (Table 5). Older age and BMI greater than 30 kg/m^2^ were significantly associated with a higher likelihood of D-DGF + ND-DGF, whilst undergoing live donor kidney transplantation was associated with a lower likelihood of D-DGF + ND-DGF.

**Table 4:** Results of multivariable logistic regression analysis of predictors of delayed graft function requiring dialysis within 72 hours of renal transplantation (D-DGF) N = 256*

**Characteristics**	**Odds ratio**	**Confidence interval**	**P value**
**Recipient characteristics**			
Statin use (yes versus no)	1.05	0.96-1.15	0.28
**Donor characteristics**			
Type			
Live versus Donation after brain death	0.12	0.03-0.43	<0.01
Donation after cardiac death versus			
Donation after brain death	10.37	3.75-28.66	<0.01
Transplant procedure characteristics			
Warm Ischemic Time (hours)	24.37	2.98-199.29	0.03

Only statin use and statistically significant variables in the final adjusted regression model are shown.

* The final variables included in the model were donor characteristics (hypertension, diabetes mellitus, smoking status, donor type, cause of death, inotropic support, estimated glomerular filtration rate [eGFR]), recipient characteristics (gender, race, BMI, diabetes mellitus, smoking status, end-stage renal failure cause, previous renal transplantation, use of statins) and operation characteristics (cold ischemic time, warm ischemic time).

**Table 5:** Results of multivariable logistic regression analysis of delayed graft function (DGF = D-DGF + ND-DGF) N = 256*

**Characteristics**	**Odds ratio**	**Confidence interval**	**P value**
**Recipient characteristics**			
Body Mass Index (kg/m^2^)			
>30 versus 18.5-24.99	6.14	2.27-16.57	<0.01
<18.5 versus 18.5-24.99	1.24	0.14-11.22	0.85
25-29.99 versus 18.5-24.99	2.03	1.00-4.13	0.05
Statin use (yes versus no)	0.98	0.39-1.06	0.59

Only statin use and statistically significant variables in the final adjusted regression model are shown.

* The initial variables included in the model were donor characteristics (body mass index [BMI], hypertension, smoking status, donor type, cause of death, inotropic support) recipient characteristics (age, , BMI, hypertension, diabetes mellitus, smoking status, end-stage renal failure cause, prior renal replacement therapy, use of statins) and operation characteristics (cold ischemic time).

Sensitivity analyses were performed with stratification by donor type. These demonstrated that statin use in living donor transplants was not significantly associated with D-DGF (p = 0.99) or clustered D-DGF + ND-DGF (OR 0.88 95% CI0.76 - 1.01; p = _0.06). Similarly, statin use in donation after brain death (DBD) transplants was not significantly associated with D-DGF (OR 1.04; 95% CI0.91 -1.18; p = 0.58) or clustered D-DGF + ND-DGF (OR 1.06; 95% CI 0.94 -1.19; p = 0.33). Multivariable logistic regression analyses were not able to be performed for the small number of kidneys donated after cardiac death (Table 1).

## Discussion

The present study is the first to examine the relationship between statin use and the occurrence of delayed graft function (DGF) following renal transplantation in humans. The key finding was that statin use by recipients prior to renal transplantation was not observed to be significantly associated with the risk of D-DGF. This finding was not altered when D-DGF and ND-DGF were pooled together.

These findings contrast with previous studies in rat models of ischemia-reperfusion injury whereby statin treatment has been found to significantly reduce the severity of acute kidney injury [16-18,21]. Statins have also have been found to protect against experimental ischemic injury to gut [19], liver [20] and lung [20] tissue. The apparent disparity in findings between these studies and ours may be potentially explained by the appreciably higher doses of statins administered in the animal models (1–10 mg/kg) and the modifying influences of immunosuppressive agents in human renal transplantation, which were not examined in the animal models. However, a previous study by our group demonstrating a significant renoprotective effect of simvastatin on cyclosporine-induced injury in primary cultures of human proximal tubule cells argues against abrogation of the renoprotective effect of statins by calcineurin inhibitors in transplantation-related ischaemia-reperfusion injury [26]. Furthermore, the beneficial effects of statins on human proximal tubule cell injury were independent of the mevalonate-cholesterol pathway [26].

Another reason for the disparity in findings may be the use of animal models for ischemia reperfusion injury. Cardinal differences between animal models and patients exist that may contribute to the differences in study outcomes [27,28]. In animal studies, statins were mostly administered through intraperitoneal and intravenous injection whereas the oral route is typically used in humans. Furthermore, in all previous studies of rat models of ischemia-reperfusion injury, the kidney was pre-treated with statins prior to ischemia or prior to reperfusion. Statin use in the donor population was not recorded in this study. We believe it would be uncommon for the donor population to be coincidentally receiving statin administration. Statin use prior to hospital admission has been reported in up to 30% of the intensive care patients [29-31]. It is unlikely that this number is representative of the donor population given their lower age and minor comorbidities [32]. Even for those donor patients that may have been on prior statin therapy, it is common practice to discontinue statins in critically ill patients because of concern regarding serious side effects [29,33]. Administration of statins to donors prior to organ retrieval (and onset of ischemic acute kidney injury) was not assessed in this study and would require separate evaluation. Indeed, early inflammatory and stress responses can be detected in donor kidneys prior to their retrieval from brain dead patients [34].

It is also possible that prior statin treatment of renal transplant recipients needed to be continued into the early post-operative period to realize any potential beneficial effects on DGF. In the present study, statin users had their statins temporarily interrupted in the immediate post-transplant period until reliable oral intake was re-established, usually around post operative day 2. The literature is divided on the risks or benefits of cessation of maintenance statin therapy in hospitalised patients. It has been shown in previous investigations that cessation of statins in certain clinical settings, such as after coronary syndromes [35], acute stroke [36] and major non-cardiac surgery [37], is associated with significantly increased morbidity and mortality. However, these findings were refuted by a recent randomized controlled trial in which the cessation of statins in patients with presumed infection was not associated with an inflammatory rebound effect or other adverse clinical consequences [31]. Given the higher incidence of ND-DGF in prior statin users (40 (43%) versus 63 (36%) P = 0.25), we consider further investigation of the role of statin withdrawal in this patient population is warranted.

The present study cannot exclude the possibility that some statins may be more effective in mitigating DGF than others. Atorvastatin was used in the majority (77%) of recipients in our study, reflecting common practice in Australia [38]. Atorvastatin, simvastatin, pravastatin and rosuvastatin all have different pharmacokinetics, including half-life time, lipophilicity and potency [39]. Although pleiotrophic effects of statins are generally considered to represent a class effect [15], most studies have shown a protective effect of statins in ischemia-reperfusion injury with simvastastin [16-18,21], whilst only one study used atorvastatin [40]. A previously published systematic review by our group of 5 randomised controlled trials found no significant effect of statin use on the risk of acute rejection in renal transplant recipients (relative risk 0.61, 95% CI 0.32-1.16) [41]. Data were not available to evaluate the effect of statins on DGF.

One of the challenges of the present study related to the definition of DGF. There are at least 18 unique definitions of DGF employed in the literature [24]. The one that is used most frequently is the need for dialysis post-transplantation, although the specified timeframe in which dialysis occurs is variable. The need for dialysis within 72 hours after transplantation is the definition used by the Australian and New Zealand Dialysis and Transplant Registry (ANZDATA; http://www.anzdata.org.au) and was therefore employed in this study. However, given that such a conservative definition potentially excludes a significant number of patients with less severe forms of DGF, a sensitivity analysis was performed to include these patients using a broader definition. Govani et al. devised and validated the creatinine reduction ratio at post-operative day 2 (CRR2) <30% as a simple, objective criterion for early diagnosis of DGF [23]. Both Rodrigo et al. and Vilar et al. subsequently demonstrated that patients with a CRR2 < 30% (ND-DGF) had a significantly lower 5 year graft survival than patients with IGF [3,4]. Nevertheless, regardless of whether the need for dialysis post-renal transplantation was considered alone or in combination with the CRR2 criterion, statin therapy was not associated with DGF in the present study.

Our study has the expected limitations of a retrospective study. Even though we adjusted for a number of patient characteristics, the possibility of residual confounding could not be excluded. Statin use in recipients was not randomized and, as such, the results could be confounded by indication bias. Statin users were significantly older with more hypertensive disease and tended to have a higher BMI, a history of prior renal transplantation and a kidney from an older donor. Such characteristics are associated with a higher incidence of DGF and could have masked any potential beneficial renoprotective effect of statins. Pre-transplant anti-hypertensive medications in both the donors and recipients were not recorded, such that a differential pre-conditioning effect of these agents on subsequent ischaemia-reperfusion injury in the statin and non-statin users could not be excluded. Since statins were only administered to recipients, the current result might only reflect an effect in the reperfusion mediated kidney injuries. Some important variables, such as recipient panel reactive antibodies, were not recorded. This was a single centre study and thus the results may not be generalisable.

## Conclusions

In conclusion, the present study did not show evidence of a significant, independent association between the use of statins in kidney transplant recipients and the occurrence of delayed graft function. Further studies on delayed graft function should examine the effects of statin pretreatment of donors (with or without recipient treatment) and the impact of continuing prior statin therapy in recipients in the immediate post-operative period.

## Competing interests

The authors declare that they have no competing interests.

## Authors’ contributions

JR was the principal investigator; conceived study; participated in design and data analysis; helped to draft manuscript; read and approved the final manuscript. DJ participated in study design and data analysis; helped to draft manuscript; read and approved the final manuscript. PK participated in study design and data analysis; helped to draft manuscript; read and approved the final manuscript. PP participated in study design and data analysis; helped to draft manuscript; read and approved the final manuscript. DW was the senior investigator overseeing the conduct of the study; participated in study design and data analysis; helped to draft manuscript; read and approved the final manuscript. All authors read and approved the final manuscript.

## Authors’ information

JR, Queensland Renal Transplant Service, Princess Alexandra Hospital, Ipswich Road, Woolloongabba, Brisbane and medical student of the University of Maastricht, Maasticht, The Netherlands.

DJ, Director of Metro South and Ipswich Nephrology & Transplant Services (MINTS), Princess Alexandra Hospital, Ipswich Road, Woolloongabba, Brisbane, Australia and Professor of Medicine and Population Health, University of Queensland, Brisbane, Australia.

PK, Deputy Director of Intensive Care, Princess Alexandra Hospital, Ipswich Road, Woolloongabba, Brisbane, Australia, and Associate Professor, University of Queensland, Brisbane, Australia.

PP, Director of Clinical Pharmacology, Princess Alexandra Hospital, Ipswich Road, Woolloongabba, Brisbane, Australia and Associate Professor, University of Queensland, Brisbane, Australia.

DW, Queensland Renal Transplant Service, Princess Alexandra Hospital, Ipswich Road, Woolloongabba, Brisbane and Associate Professor, University of Queensland, Brisbane, Australia.

## Pre-publication history

The pre-publication history for this paper can be accessed here:

http://www.biomedcentral.com/1471-2369/13/111/prepub
